# Reversibility of liver fibrosis

**DOI:** 10.1186/1755-1536-5-S1-S26

**Published:** 2012-06-06

**Authors:** Antonella Pellicoro, Prakash Ramachandran, John P Iredale

**Affiliations:** 1 Centre for Inflammation Research, Queen's Medical Research Institute, University of Edinburgh, Edinburgh, UK

## Abstract

Liver fibrosis, and its end stage cirrhosis are a major cause of morbidity and mortality and therapeutic options are limited. However, the traditional view of liver disease as an irreversible process is obsolete and it is now evident that the development of liver fibrosis is a dynamic and potentially bidirectional process. Spontaneous resolution of scarring is seen in animal models of liver fibrosis and in human trials in which the stimuli responsible for chronic or repeated hepatic inflammation is successfully removed. Key players in the process are hepatic stellate cells, macrophages, MMPs and their inhibitors Timps. It is also evident that in advanced fibrotic liver disease, specific histological features define what is currently described as "irreversible" fibrosis. This includes the development of paucicellular scars enriched in extensively cross-linked matrix components, such as fibrillar collagen and elastin. Our recent work has focused on the role of macrophage metalloelastase (MMP-12) in the turnover of elastin in reversible and irreversible models of fibrosis. We have shown that elastin turnover in liver injury and fibrosis is regulated by macrophages via Mmp-12 expression, activity and ratio to its inhibitor Timp-1. Failure of elastin degradation, together with increased deposition leads to accumulation of elastin in the fibrotic scars.

## Introduction

The complications of liver fibrosis represent a major health burden to our society. World-wide, chronic viral hepatitis is the predominant cause of liver fibrosis, whereas in the Western Hemisphere alcoholic liver disease, chronic viral hepatitis and increasingly non-alcoholic fatty liver disease are the main causes. Furthermore the prevalence of liver cirrhosis in the UK has increased dramatically in the past two decades [1] and it is now the fifth cause of death . Liver fibrosis and its end-stage, cirrhosis, result from a sustained wound healing response to chronic or repeated injury, leading to formation of scar tissue, loss of tissue architecture and organ failure. Regardless if the initial cause of injury, fibrosis represents the final common pathway of chronic hepatic inflammation. However, the wound healing response is a dynamic process and has the potential to resolve without scarring. Removal of the injury stimulus has been shown to improve liver fibrosis in alcoholic liver disease [2], viral hepatitis [3-5], biliary obstruction [6] and autoimmune hepatitis [7]. Current treatments for liver fibrosis are directed towards suppression or removal of inflammatory stimuli that drives the development of fibrosis. However for severe end-stage liver disease, orthotopic liver transplantation is the only effective treatment but the availability, suitability and long-term effectiveness of liver transplantation severely restrict it utility as therapy.

Experimental models have confirmed the possibility of reversion of established liver fibrosis [8-10]. Rats injured with carbon tetrachloride for 4 to 8 weeks develop fibrosis, but after cessation of the injury are able to revert to virtually normal morphology. On the other hand, when the injury is protracted for longer time, (12 weeks) cirrhosis develops and in the absence of ongoing injury there is only partial reversal with remodelling of a micro-nodular to a macro-nodular architecture [10]. It is therefore apparent that liver cirrhosis is not simply an extended form of liver fibrosis, but is associated with particular architectural and biochemical features that may limit the potential for resolution.

One of the first events in liver fibrosis is the activation of resident innate inflammatory cells and the recruitment of additional inflammatory cells. These include recruited monocytes/macrophages, with further contributions from other cell lineages in specific disease states. Many inflammatory mediators are produced by damaged hepatocytes, cholangiocytes, endothelial and inflammatory cells. The activation of hepatic myofibroblasts, including hepatic stellate cells (HSCs) is a critical step in the interlinked processes of tissue injury and regeneration. HSCs normally reside in the perisinusoidal space where their principal function is the storage of retinoids [11,12]. Following liver injury HSCs become activated (transdifferentiate) into contractile and proliferative myofibroblasts that are responsible for secreting much of the ECM that characterises liver fibrosis. In early fibrosis there is accumulation of fibronectin and collagen types III and IV [13]. As liver fibrosis progresses, the ECM accumulates increasing amounts of types I and type IV fibrillar collagens, addition of other matrix components including elastin. Both increased matrix deposition and reduction in matrix degradation concur in the accumulation of the fibrotic scar [8]. Activated HSCs up-regulate the expression of potent inhibitors of metalloproteinases (TIMPs) [14]. Consequently the balance between the action of matrix-degrading metalloproteinases (MMPs) and their inhibitors is shifted, facilitating matrix accumulation. Similarly, the resolution of liver fibrosis is accompanied by HSC apoptosis, which simultaneously removes both an important source of ECM deposition and MMP inhibition [15]. Importantly, there is a close anatomical correlation between the site of initial injury and the subsequent deposition of ECM. However, as fibrosis progresses bridging fibrosis develops, resulting in formation of the fibrous septae and regenerative nodules that characterise cirrhosis. What are the features of cirrhosis that prevent successful remodelling? In animal models of cirrhosis, MMPs are present but their action is inhibited by high levels of TIMP-1 and to a lesser extent TIMP-2 [8-10]. Interestingly, in a rat CCl_4 _model, TIMP-1 over-espressing rat develop the same level of fibrosis as wild type, but are unable to resolve after removal of the injury. In addition, in these animals hepatic stellate cell apoptosis is decreased [16]. In our rodent model, while more recently deposited septae are readily degraded, the older septae appear more resistant to degradation [10]. These pauci-cellular scars are particularly rich in elastin and cross-links that may render them more resistant to MMP-mediated degradation.

We have recently shown that the accumulation of elastin is, rather than being only the result of excessive secretion, also results from a failure of elastin degradation [37]. In a rat model of fibrosis, both tropoelastin and macrophage metallo-elastase (MMP-12) are increased in active fibrosis. Elastin is strongly expressed from the onset of injury but, in contrast to collagen I, only accumulates late, suggesting that degradation occurs during the early phases of injury. On the other hand a higher TIMP-1/Mmp-12 ratio is observed in early vs established fibrosis. In addition, Mmp-12 knock-out mice have both increased collagen and elastin deposition in a TAA model of cirrhosis. Current studies are aimed to evaluate the reversibility of fibrosis in this model.

The role of the ECM in promoting HSC survival should not be understated. Cell matrix components including collagen-1 [17] and interactions mediated through integrins [18,19] and discoidin domain receptors [20] can promote HSC activation and promote HSC survival. Furthermore, there is increasing evidence that the altered mechanical properties of fibrotic ECM may directly facilitate myofibroblast activation, proliferation and survival through mechanotransduction responses consequent to enhanced cytoskeletal tension of cells exposed to stiff fibrotic matrices [21-24]. In addition, cellular responses to increased liver stiffness in acute inflammation (oedema) may represent a conserved mechanism linking acute liver injury to wound healing and regeneration [25,26].

Resident and recruited inflammatory cells have complex effects on both the development of fibrosis and its subsequent resolution [27]. Prominent amongst these are recruited monocytes/macrophages and the liver-resident Kupffer cells. Macrophages have long been regarded as leading protagonists in the development of tissue fibrosis. The inhibition of macrophage migration by adenoviral over-expression of a dominant-negative form of macrophage chemotactic protein-1 (MCP-1) has been shown to reduce macrophage influx and ameliorate fibrosis in a rodent model of liver fibrosis [28]. However, it has subsequently been demonstrated that macrophages have distinct roles in the injury and recovery phases of liver fibrosis [29]. This was achieved in experiments with a transgenic mouse (CD11b-DTR) in which macrophages could be selectively depleted during both the injury and recovery phases that follow carbon tetrachloride administration in mice. Macrophage depletion at the time of injury resulted in reduction in both scarring and myofibroblast numbers. In contrast, macrophage depletion during recovery from fibrosis led to a reduction in scar resolution. This suggested that distinct subpopulations of macrophages have discrete functions in the injury and recovery phases of liver fibrosis.

Macrophages are known to be an important source of pro-fibrotic chemokines. Macrophage-conditioned media has been shown to promote HSC activation in vitro. This effect is mediated by transforming growth factor-beta (TGFβ) and the induction of platelet-derived-growth-factor (PDGF) receptors on HSCs [30]. Conversely, macrophages may promote HSC apoptosis during the resolution of liver fibrosis through expression of HSC death ligands, including TNFα-related apoptosis-inducing ligand (TRAIL) and MMP-9 [18,31,32]. Macrophages also appear to function more directly in matrix remodelling through production of macrophage-derived metalloproteinases, including MMP-9 [33] and MMP-13 [34]. Scar-associated macrophages have been shown to be the principal source of MMP-13 in a rodent model of liver fibrosis [35]. Furthermore MMP-13 expression is restricted to regions of liver fibrosis rich in scar-associated macrophages. This has important implications in relation to the failure of ECM-degradation in the paucicellular elastin-rich scars that characterise advanced cirrhosis. Could macrophages or stem cell technology be harnessed to seed the hepatic scar with cells that may facilitate successful remodelling? The results of provisional experiments are certainly encouraging.

The role of other cells of the innate and adaptive immune system in the development of liver fibrosis is less well defined and has been reviewed in detail elsewhere [27]. Recent years have seen renewed interest in the role of natural killer (NK) cells in the resolution of liver fibrosis. NK cells have been shown to facilitate HSC deletion in liver fibrosis [36]. NK cell activation with polyinosinic-polycytidylic acid or interferon-gamma has been shown to induce cell death in activated stellate cells and reduce the severity of scarring in two murine models of liver fibrosis. Activated HSCs express high levels of retinoic acid early inducible-1, a ligand for the NKG2D receptor found on NK cells. It was demonstrated that the enhanced cytotoxicity of NK cells against activated HSCs was a consequence of up-regulation of both the NKG2D receptor and the TRAIL death ligand. An important implication of this study is that the manipulation of NK cells might represent a practical therapeutic strategy to facilitate HSC deletion in liver fibrosis.

## Discussion

Current evidence indicates that the development of liver fibrosis is a dynamic and potentially bidirectional process. Spontaneous resolution of scarring is readily seen in animal models of liver fibrosis and in human trials in which the stimuli responsible for chronic or repeated hepatic inflammation is successfully removed. However, it is also evident that in advanced fibrotic liver disease, specific histological features define what our group has described as "irreversible" fibrosis. This includes the development of paucicellular scars enriched in extensively cross-linked elastin and fibrillar collagen. In the decades to come a key priority must be the development of strategies to facilitate the repopulation and effective remodelling of areas of advanced scarring. It is likely that this will require a combination of cellular therapies and the development of pharmaceutical agents addressing multiple targets in pathways involved in fibrogenesis.

## Competing interests

The authors declare that they have no competing interests.
